# Isolation and functional characterization of a glucose-6-phosphate/phosphate translocator (*IbG6PPT1*) from sweet potato (*Ipomoea batatas* (L.) Lam.)

**DOI:** 10.1186/s12870-021-03372-0

**Published:** 2021-12-16

**Authors:** Zhengdan Wu, Zhiqian Wang, Kai Zhang

**Affiliations:** College of Agronomy and Biotechnology, Southwest University, Beibei, Chongqing, 400715 P. R. China

**Keywords:** Sweet potato, Glucose-6-phosphate/phosphate translocator, Starch, Carbohydrate metabolism

## Abstract

Sweet potato (*Ipomoea batatas* (L.) Lam.) is a good source of carbohydrates, an excellent raw material for starch-based industries, and a strong candidate for biofuel production due to its high starch content. However, the molecular basis of starch biosynthesis and accumulation in sweet potato is still insufficiently understood. Glucose-6-phosphate/phosphate translocators (GPTs) mediate the import of glucose-6-phosphate (Glc6P) into plastids for starch synthesis. Here, we report the isolation of a GPT-encoding gene, *IbG6PPT1*, from sweet potato and the identification of two additional *IbG6PPT1* gene copies in the sweet potato genome. *IbG6PPT1* encodes a chloroplast membrane–localized GPT belonging to the GPT1 group and highly expressed in storage root of sweet potato. Heterologous expression of *IbG6PPT1* resulted in increased starch content in the leaves, root tips, and seeds and soluble sugar in seeds of *Arabidopsis thaliana*, but a reduction in soluble sugar in the leaves. These findings suggested that *IbG6PPT1* might play a critical role in the distribution of carbon sources in source and sink and the accumulation of carbohydrates in storage tissues and would be a good candidate gene for controlling critical starch properties in sweet potato.

**Supplementary Information:**

The online version contains supplementary material available at 10.1186/s12870-021-03372-0.

## Background

Sweet potato (*Ipomoea batatas* (L.) Lam.) is an important food crop that is cultivated in over 100 countries due to its stable yield, rich nutrient content, low input requirements, multiple uses, high yield potential, and adaptability under a range of environmental conditions [32, 34, 36, 39]. Sweet potato is grown mainly for its edible, starchy storage root, which is 50–80% starch by dry matter [38]. This high starch content renders sweet potato a good source of carbohydrates, an excellent raw material for starch-based industries, and a strong candidate as an inexpensive raw material for biofuel production [12, 20, 27]. Starch is synthesized in plants through a complex pathway involving multiple enzymes and transporters [17, 25, 36]. In recent decades, more and more researches on the sweet potato were focused on increasing the starch accumulation by regulating starch biosynthesis related genes in the storage root, such as *IbGBSSI*, *IbSBE*, *IbSRF*, *IbSnRK1*, *IbAATP*, *IbEXP1* [13]. However, the molecular basis of starch biosynthesis and accumulation in sweet potato is still insufficiently understood.

Starch biosynthesis begins with the synthesis of sucrose, the important product of photosynthesis, in source tissues. During this process, sucrose can be converted to glucose-6-phosphate (Glc6P) and then imported into the plastid by glucose-6-phosphate/phosphate translocators (GPTs), the proteins belonging to the transporter subfamily of phosphate translocators (PTs). Three classes GPTs have been identified in plants and shouwn to play important roles in several physiological processes [2]. In Arabidopsis, *GPT1* is essential for the development of male and female gametophytes, embryos, and seeds [3, 35]. In other plants, GPT1 also plays a major role in the regulation of starch synthesis. In Narbonne vetch (*Vicia narbonensis*), GPT1 is critical for starch synthesis and storage in developing seeds. In *Vicia* transgenic plants expressing antisense GPT1 via *Agrobacterium*-mediated transformation, amyloplasts developed later and were smaller in size, starch biosynthesis was reduced, and storage protein biosynthesis increased [24]. In rice, pollen grains from homozygous *osgpt1* mutant plants fail to accumulate starch granules, resulting in pollen sterility [23]. By contrast, in Arabidopsis, *GPT2* is expressed when photosynthesis is increased by light, which allows increased net import of Glc6P from the cytosol to chloroplasts, thus facilitating starch synthesis during stochastic high-light conditions [5, 28]. *GPT2* responds rapidly to glucose and sucrose and plays an essential role in interpreting environmental signals [3, 28]. In tobacco (*Nicotiana tabacum*), *GPT3* could allow accumulating cytosolic glucose-6-phosphate to return to the chloroplast. This could feed starch synthesis or a glucose-6-phosphate shunt in the Calvin cycle [2]. However, the role of GPTs in sweet potato has not been investigated.

In our previous work, the comparative transcriptome analysis results showed that a sweet potato *GPT* gene, showed expression patterns during storage root development and among sweet potato genotypes with different starch properties. This gene was strongly expressed in the storage roots of sweet potato at 65, 80, 95, 110, 125 days after transplanting (DAP), and the expression level in high starch content varieties was higher than that in low starch content varieties, indicating this GPT gene is probably involved in starch properties regulation in sweet potato [36]. Here, we cloned this *GPT* gene and analyzed its protein localizations, sequence features, and functions. Our results provide important insights into the mechanisms underlying the starch properties of sweet potato.

## Results

### Two GPT-encoding genes were cloned from sweet potato

**Fig. 1:**
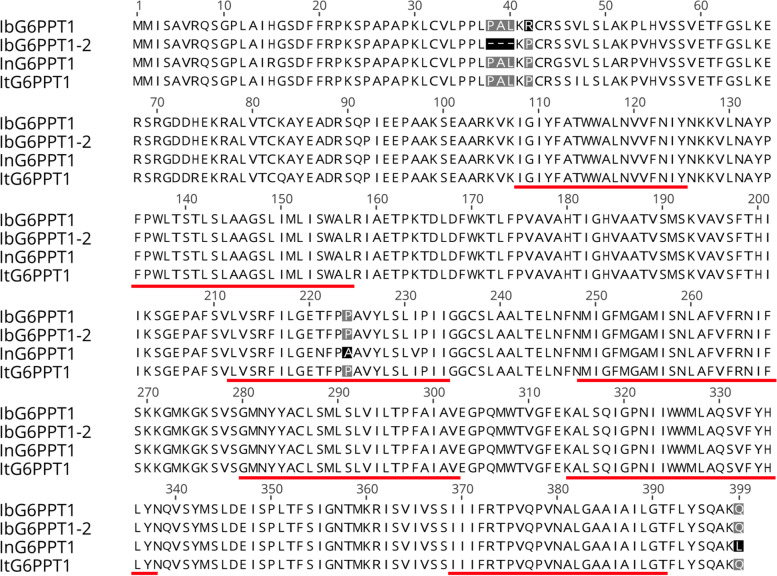
Alignment of IbG6PPT1, IbG6PPT1-2, and *Ipomoea* genus GPT1 proteins. ItG6PPT1, *Ipomoea triloba* GPT1 (XP_031105621.1); InG6PPT1, *Ipomoea nil* G6PPT1 (XP_019193616.1). The amino acids underlined in red form transmembrane helixes based on prediction using TMHMM; black and grey highlighting indicate amino acid differences between the species

To ensure that the full-length mRNA sequence of sweet potato *GPT* genes could be obtained, the RACE method was used for cloning. Two cDNA sequences encoding the target sweet potato *GPT* gene were obtained, named *IbG6PPT1* and *IbG6PPT1-2*. The obtained full-length mRNA sequences were 1767 and 1763 nt in size, corresponding to 1200 and 1191 bp of ORFs and encoding 400-aa and 397-aa protein sequences, respectively. The two genes shared 96.627, 98.083, and 98.747% identity at the mRNA, CDS, and putative amino acid levels, respectively. The two proteins differed in only five amino acids (Fig. 1), including a deletion of the L_37_P_38_A_39_ sequence in the shorter GPT.

### Sweet potato has a third IbG6PPT1-like gene

The sweet potato genome is annotated with three *IbG6PPT1* gene members: *IbG6PPT1* located on pseudochromosome 3 (chr3), *IbG6PPT1-2* located on chr2, and another *IbG6PPT1*-like gene also expected to be located on chr2. However, the sequence of this *IbG6PPT1*-like gene was not cloned from our cDNA library. Amino acid differences between IbG6PPT1 and IbG6PPT1-2 were not located at conserved domains or important transmembrane domains, indicating that these proteins are likely functional.

### The cloned GPT genes belong to the GPT1 group

**Fig. 2:**
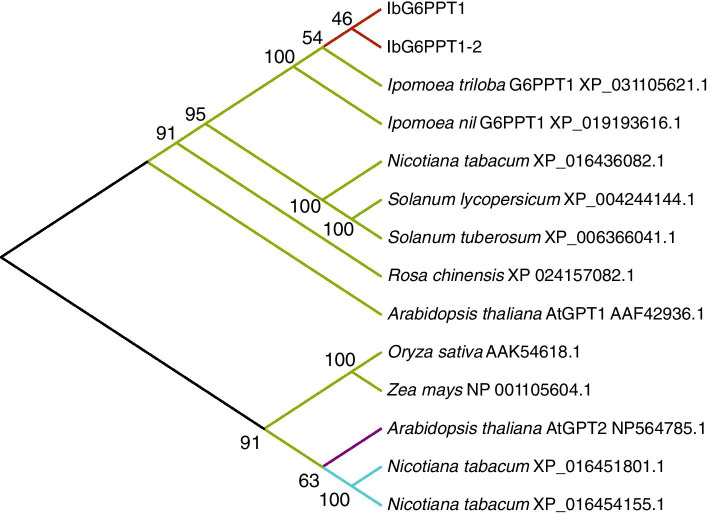
Phylogenetic analysis of GPT proteins. The phylogenetic tree was constructed using the Neighbor-Joining method implemented in MEGA-X software. The numbers on the branches are bootstrap values (based on 1000 repeats). Red lines represent IbG6PPT1 and IbG6PPT1-2, green lines represent the GPT1 group, light purple lines represent the GPT2 group, cyan lines represent the GPT3 group

The GPT subfamily includes three groups: GPT1, GPT2, and GPT3 [2]. The sweet potato *GPT* genes showed 98.75 and 97.99% identity with *Ipomoea nil* and *Ipomoea triloba* GPT1, respectively. A GPT phylogenetic tree showed that the GPT1 group consisted of two sweet potato GPT proteins as well as *Ipomoea trilobal*, morning glory (*Ipomoea nil*), tobacco (*Nicotiana tabacum*), potato (*Solanum tuberosum*), tomato (*Solanum lycopersicum*), China rose (*Rosa chinensis*), *Arabidopsis thaliana*, rice (*Oryza sativa*), and maize (*Zea mays*) GPT1 proteins. The GPT2 group consisted of AtGPT2, whereas the GPT3 group mainly consisted of two *N. tabacum* GTP3 proteins, XP_016451801.1 and XP_016454155.1 [2] (Fig. 2). Therefore, the obtained sweet potato GPTs belong to the GPT1 group.

The protein sequence alignment and phylogenetic tree analysis showed that IbG6PPT1 was more similar than IbG6PPT1-2 to GPT1 proteins from *Ipomoea nil* and *Ipomoea triloba* (Figs. 1 and 2), indicating that *IbG6PPT1* might match previous GPT1 findings better in the *Ipomoea* genus. Thus, we focused on *IbG6PPT1* for the remainder of this work.

### IbG6PPT1 is likely a chloroplast-located GPT

**Fig. 3:**
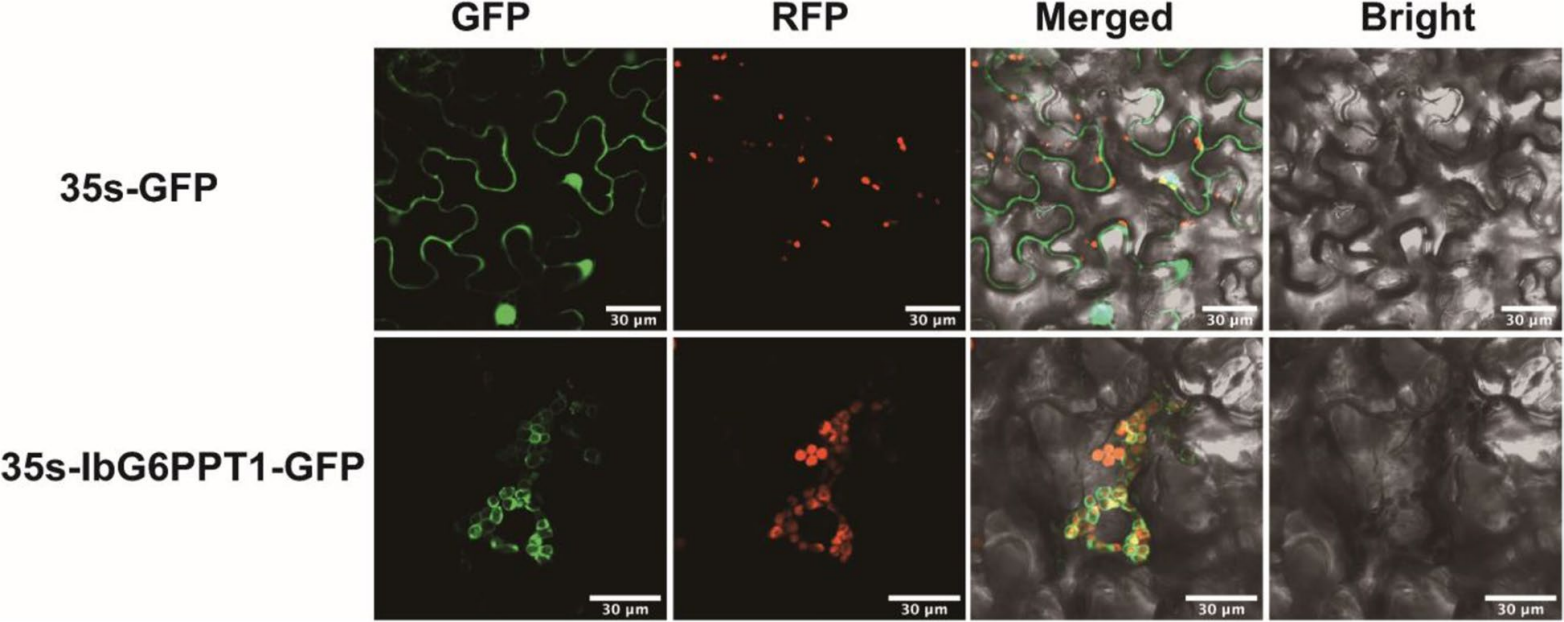
IbG6PPT1 localizes to the chloroplast membrane in *N. benthamiana* leaf. GFP: green fluorescent protein. RFP: chloroplast marker. Merged: combined GFP and RFP signals. Bright: bright field. Bars: 30 μm

**Fig. 4:**
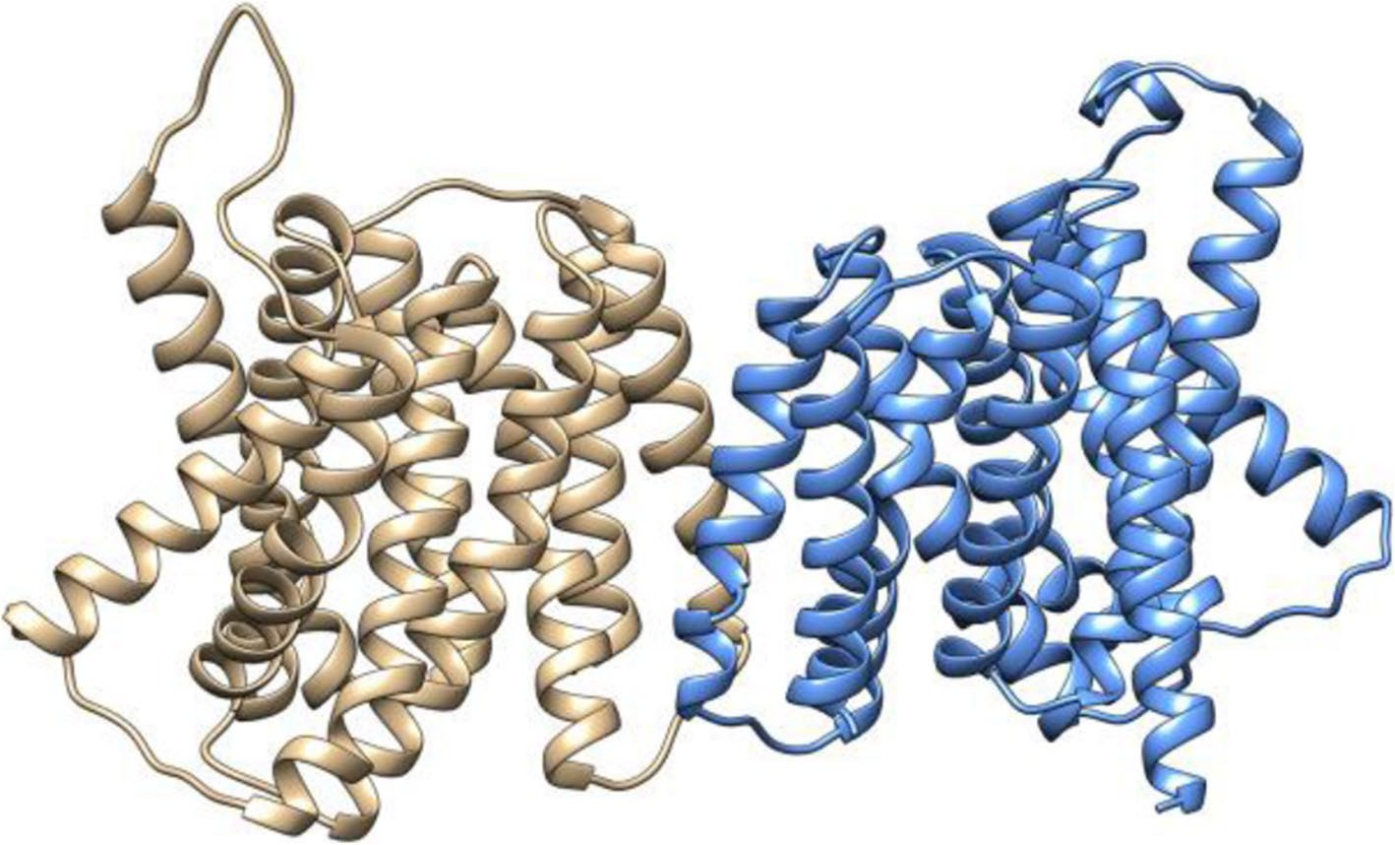
Predicted three-dimensional structure models of IbG6PPT1. Two IbG6PPT1 proteins (shown in yellow and blue, respectively) form a dimer

We constructed a vector expressing the IbG6PPT1 protein with a GFP-tagged and transiently expressed it in *Nicotiana benthamiana*. The IbG6PPT1-GFP signal surrounded the chloroplast marker fluorescence, indicating that IbG6PPT1 localizes to the chloroplast membrane, whereas the control signals was located on the nucleus and plasma membrane in *N. benthamiana* plants (Fig. 3). Signal peptide analysis indicated that IbG6PPT1 is a non-secreted protein. TMPred and TMHMM prediction showed that IbG6PPT1 has seven transmembrane domains (Fig. 1), indicating that IbG6PPT1 proteins are chloroplast membrane bound and have an active role in Glc6P transport across the chloroplast membrane. Modeling of the three-dimensional (3D) structure of IbG6PPT1 predicted that two IbG6PPT1 proteins form a homodimer (Fig. 4). In addition, IbG6PPT1 contains a conserved sugar phosphate transporter domain [11]. These results strongly suggest that IbG6PPT1 is a chloroplast membrane–localized protein in sweet potato.

### IbG6PPT1 is highly expressed in sweet potato storage root

**Fig. 5:**
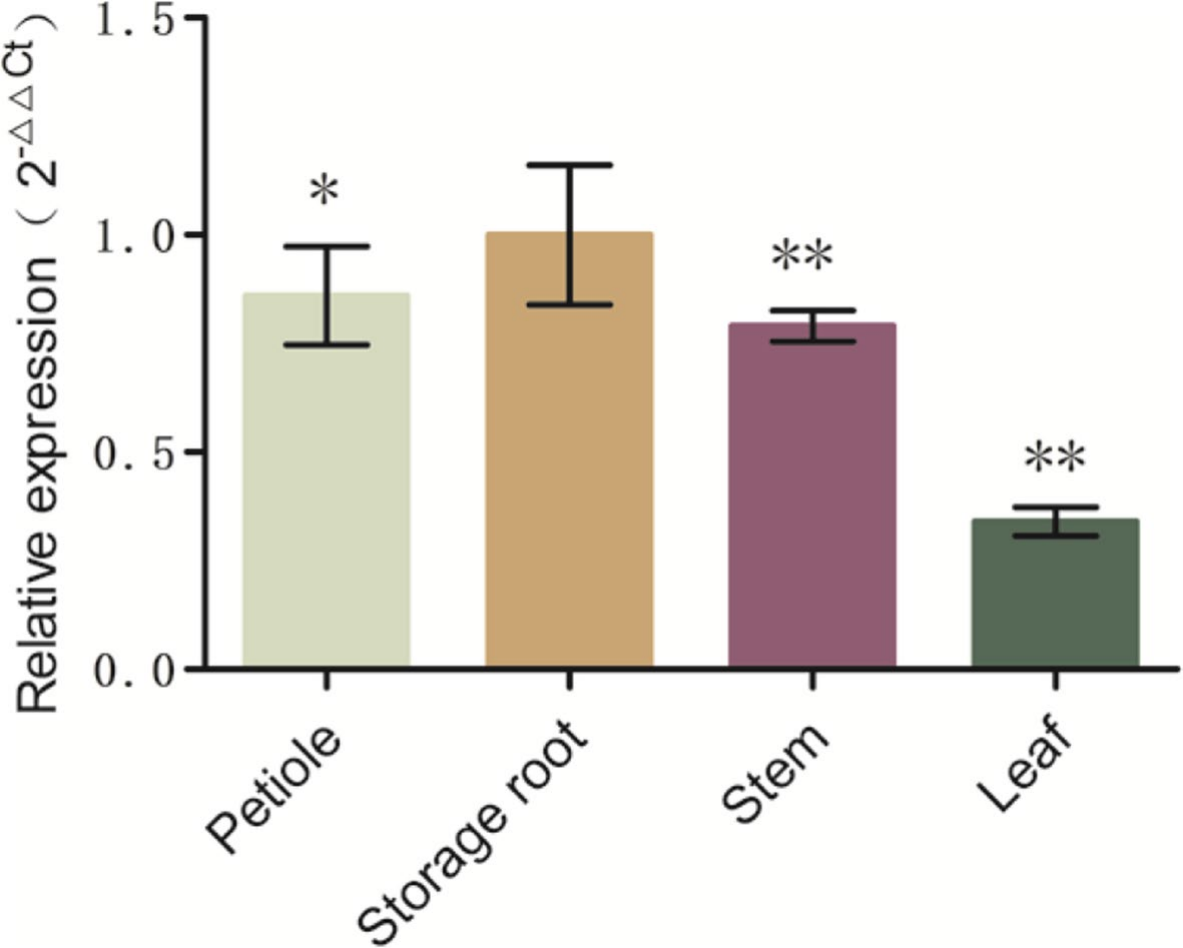
Expression of *IbG6PPT1* in the petiole, storage root, stem, and leaf of the sweet potato variety Xushu 22, as determined by qRT-PCR. Each value is the mean ± SE of at least three independent measurements. “*” represents *P* value < 0.05, “**” represents *P* value < 0.01, and “***” represents *P* value < 0.001

In order to detect the expression pattern of *IbG6PPT1* for different tissues in sweet potato, qRT-PCR was used to analyzed the expression of *IbG6PPT1* in petiole, stem, leaf and storage root. *IbG6PPT1* was expressed in all tissues but showed its highest expression in storage roots, followed by the petiole, stem, and leaf (Fig. 5). Interestingly, *IbG6PPT1* showed the higher expression in storage root than in leaf.

### Heterologous expression of IbG6PPT1 affects starch and sugar content

**Fig. 6:**
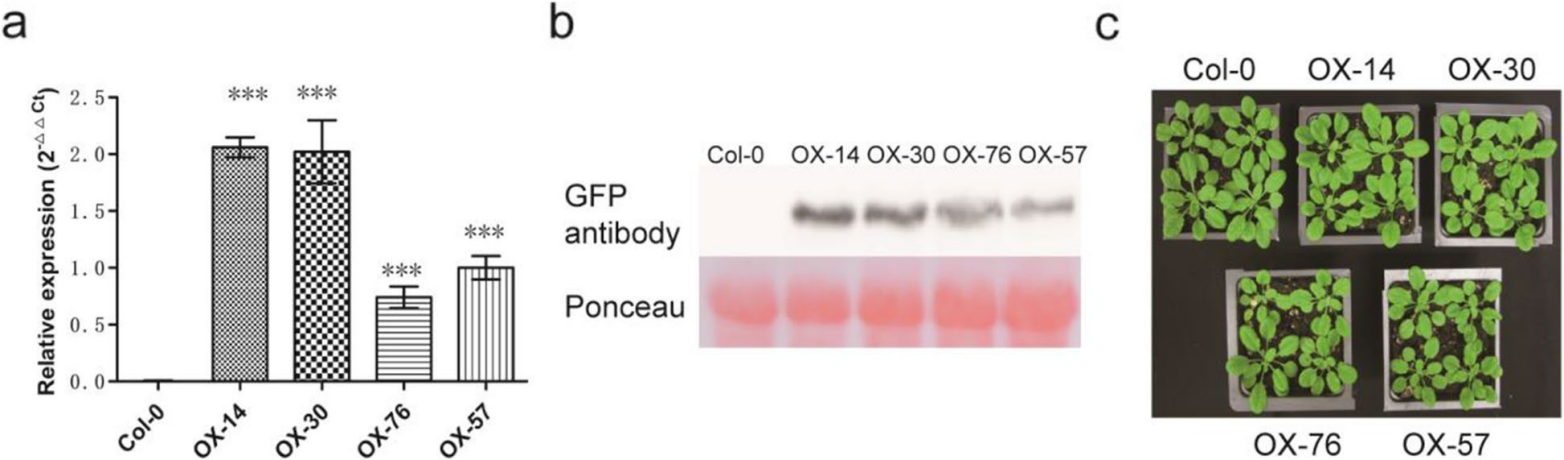
Heterologous expression of *IbG6PPT1* in *A. thaliana.***a** qRT-PCR detection of *IbG6PPT1* expression; each value is the mean ± SE of at least three independent measurements. **b** Western bloting detection of IbG6PPT1. **c** Phenotype of 4-week-olds *A. thaliana* plants heterologously expressing *IbG6PPT1*. Col-0, control plants; OX-14, OX-30, OX-76, and OX-57, four transgenic lines. “*” represents *P* value < 0.05, “**” represents *P* value < 0.01, and “***” represents *P* value < 0.001

In order to accelerate the functional analysis of IbG6PPT1, we transformed a *p35S*::*IbG6PPT1-YFP* construct into wild-type (Col-0) *A. thaliana*. Four independent homozygous transgenic lines, designated OX-14, OX-30, OX-76, and OX-57, were selected from the T2 progeny and used for further detection. Analysis of *IbG6PPT1-YFP* expression by qPCR and western blotting showed that the fusion protein was heterologously expressed in these transgenic lines (Fig. 6a and b). There were no differences in growth and development between the transgenic progeny and the wild-type control (Fig. 6c).

**Fig. 7:**
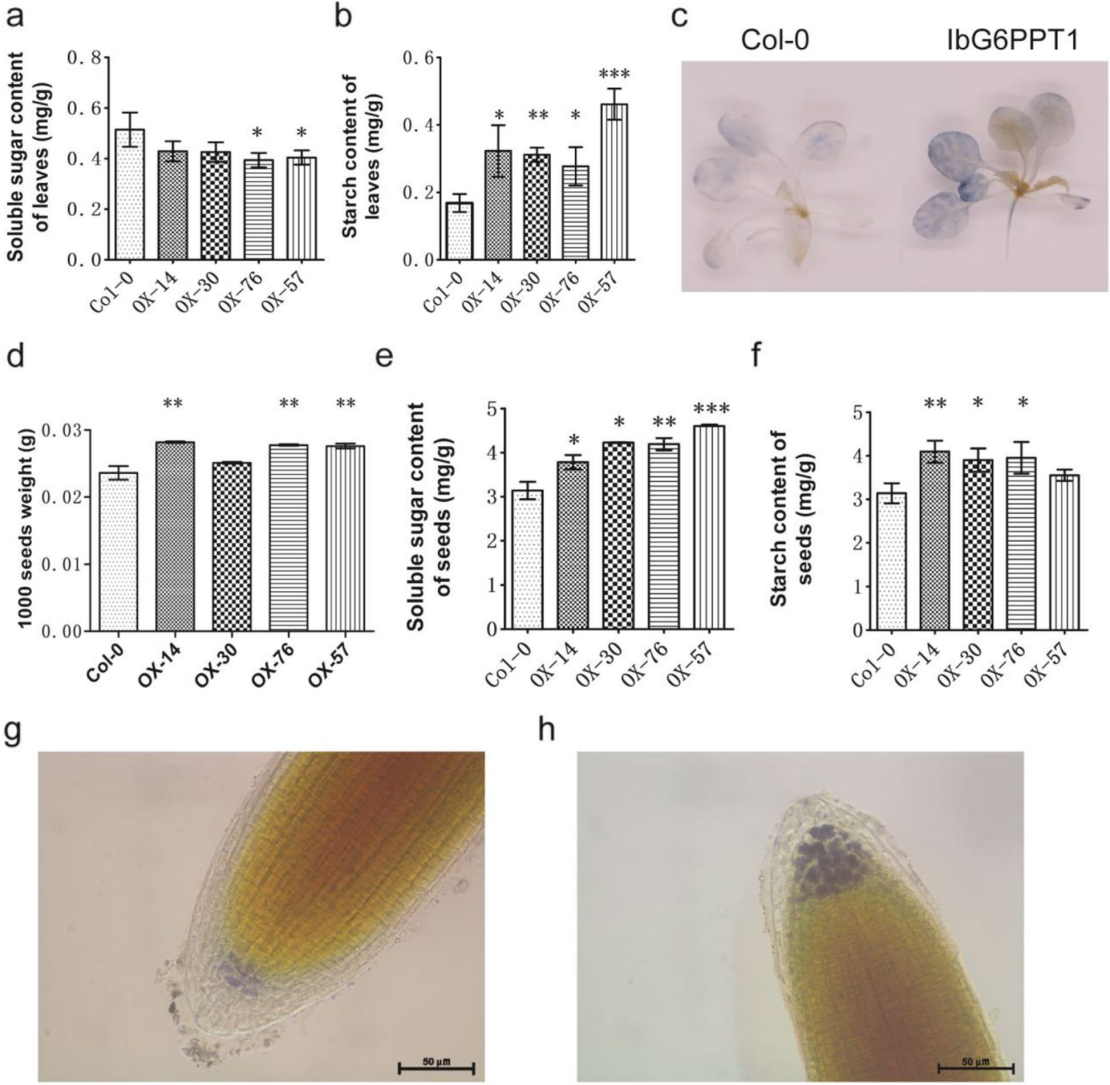
Heterologous expression of *IbG6PPT1* in *A. thaliana* alters the starch and soluble sugar content of the plants. **a** Soluble sugar content and **b** starch content of the leaves of 6-week-old *A. thaliana* plants. **c** Iodine-stained starch in the leaves of 3-week-old seedings. **d** 1000 seeds weight, **e** soluble sugar content and **f** starch content in the seeds of the transgenic and control plants. **g** and **h** Iodine-stained starch in the root tips of control and transgenic plants, respectively. Bars: 50 μm. Col-0, control plants; OX-14, OX-30, OX-76, and OX-57, four transgenic lines. Each value is the mean ± SE of at least three independent measurements. “*” represents *P* value < 0.05, “**” represents *P* value < 0.01, and “***” represents *P* value < 0.001

In contrast to their wild-type-like appearance, the soluble sugar content in the leaves of the transgenic lines was only 76.59–83.40% of control (Fig. 7a, Table S1). Meanwhile, the leaves of the 6-week-old transgenic plants had a 1.65- to 2.75-fold higher measured starch content than the control (Fig. 7b, Table S1), which was confirmed by iodine staining in 3-week-old seedlings (Fig. 7c). Surprisingly, the 1000 seed weights of the transgenic lines were 1.06- to 1.19-fold higher than in the control plants (Fig. 7d, Table S1). Further analyses showed that the soluble sugar content and starch content in the seeds of transgenic *IbG6PPT1-YFP* lines were 1.20- to 1.47-fold and 1.13- to 1.31-fold higher than in the control plants, respectively (Fig. 7e and f, Table S1). In the root tips, iodine staining showed that the starch content of transgenic lines was higher than that in control plants (Fig. 7g and h). Above all, heterologous expression of the *IbG6PPT1* gene altered soluble sugar and starch content in the leaves, and increased both starch and soluble sugar contents in the seeds of *A. thaliana*.

## Discussion

### IbG6PPT1 is present in several gene copies that may have different functions

The sweet potato genome is allohexaploid (2*n* = 6*x* = 90), containing two B1 and four B2 component genomes (B1B1B2B2B2B2) [8, 33, 35]. Therefore, there may be up to six copies of each gene. In this study, we cloned two *GPT1* genes that share a high level of identity in both the mRNA and protein sequences (Fig. 1). However, we found three potential *IbG6PPT1* genes in the genome database, the two we cloned and another one on chr2 that might be a homolog or paralog of one of the cloned genes. During the evolution of sweet potato’s polyploid genome, the duplicated genes might have developed expressional, regulatory, or functional divergence [7, 30]. Because of the very high sequence similarity between the *IbG6PPT1* genes, it is difficult to examine the expression pattern or function of a single such gene. Future work should investigate whether the three *IbG6PPT1* genes show functional divergence in Glc6P transport and thus play different roles in starch accumulation and sugar metabolism in sweet potato. Alternately, they may not have diverged as strongly, and one gene’s function may have been compensated for by the function of another gene. Future genetic engineering of the sweet potato will require gene function studies to determine the contribution of each gene copy to relevant phenotypes and identify the major gene controlling sweet potato starch properties.

### IbG6PPT1 has similar functions to other GPT1 proteins

GPT1 proteins transport Glc6P into plastids for fatty acid and/or starch biosynthesis, depending on the plant species [37]. In *A. thaliana*, fatty acid biosynthesis in pollen is controlled by regulating *AtGPT1* expression through the MKK4/MKK5-MPK3/MPK6 cascade and the downstream transcription factors WRKY2 and WRKY34 [37]. GPT1 is also essential for starch biosynthesis in Narbonne vetch and rice [23, 24]. Starch is the major carbon storage molecule of sweet potato, represents more than half of dry matter in the storage root, the organ that determines sweet potato’s economic value as a crop, whereas fatty acids are almost undetectable. Expression of *IbG6PPT1* was higher in the storages roots of high starch contents varieties than in low starch contents varieties [36], which suggested *IbG6PPT1* is critical for starch biosynthesis in sweet potato. Like *AtGPT1*, which is expressed ubiquitously throughout *A. thaliana* development [21], we found that *IbG6PPT1* is expressed in both aboveground and underground organs in sweet potato (Fig. 5), suggesting potential functions in Glc6P transport in both autotrophic tissues and heterotrophic tissues. Interestingly, the higher expression of *IbG6PPT1* in roots than in leaves suggests that it may function in non-green tissues rather than in photosynthetic tissues. The localization of IbG6PPT1 to the chloroplast membrane (Fig. 3) implied that it may function in transporting Glc6P from the cytosol into plastids.

To better elucidate the function of the *IbG6PPT1* gene in starch accumulation, we cloned *IbG6PPT1*, and heterologously expressed it in *A. thaliana*, and then measured starch accumulation in the resulting transgenic plants. *IbG6PPT1* expression increased starch accumulation in *A. thaliana* leaves, seeds, and root tips, suggesting that it promotes starch biosynthesis (Fig. 7).

It was reported that in Arabidopsis, *GPT1* is highly expressed at the late stages of pollen development, where it drives Glc6P from the cytosol and into plastids for fatty acid biosynthesis, and thus plays an important role in lipid body biogenesis during pollen maturation [37]. Lipid bodies and protein are the major storage compounds in mature *A. thaliana* seeds, each accounting for up to 40% of the dry weight [1], whereas starch are lower. The 1000 seeds weight we observed in *IbG6PPT1-*expressing plant is greater than control plant, indicating that *IbG6PPT1* may also promote storage matter accumulation in *A. thaliana* seeds.

### IbG6PPT1 enhances transport activity from sink to source and promotes carbohydrate accumulation in *A. thaliana* storage tissues

Sucrose is a major end product of photosynthesis and the primary sugar transported within plants [31]. In heterotrophic tissues, sucrose imported from photosynthetic tissues is converted to Glc6P, and some Glc6P can be transported into the plastid through GPTs for starch and/or fatty acid biosynthesis. Another portion of the Glc6P is metabolized in the cytosol to phosphoenolpyruvate (PEP), which is essential for the biosynthesis of lipids and other storage substances [18]. In *IbG6PPT1*-expressing *A. thaliana*, the starch content in the leaves increased significantly, while the soluble sugar content was reduced, compared to that in control plants (Fig. 7). Thus, heterologous expression of *IbG6PPT1* promoted starch accumulation and sugar metabolism, probably due to the high expression of GPT, which would be expected to increase the level of Glc6P imported into the chloroplast or amyloplast for starch synthesis. Compared with control, the starch content and soluble sugar content were increased in seeds of *IbG6PPT1*-expressing *A. thaliana*. This is probably caused by heterologous expression of *IbG6PPT1* in *A. thaliana* promote carbohydrate transferred from sources to sink and thus contribute to the observed carbohydrate accumulation in transgenic seeds compared with controls. This conclusion was further illustrated by the decreased of soluble sugar content in leaves and increased of starch content in roots of *IbG6PPT1*-expressing *A. thaliana* compared with control.

It also should be pointed out that *IbG6PPT1* is highly expressed in the transgenic *A. thaliana* plants, but the substance that could be translocated was limited. Thus, although *IbG6PPT1* expressed higher in the lines OX-14 and OX-30 than in OX-76 and OX-57, no more sugar and starch content change was observed in OX-14 and OX-30. It’s worthy to further investigate the potential of *IbG6PPT1* in promoting starch accumulation and sugar metabolism in the crops accumulating high level of photosynthetic products.

## Conclusion

In conclusion, our data indicates heterologous expression of *IbG6PPT1* increased the starch content in the leaves, seeds, and root tips in *A. thaliana*, but did not affect the growth and development of transgenic plants, suggesting the utilization potential of *IbG6PPT1* in promoting starch accumulation in other crops. Moreover, *IbG6PPT1* might plays a critical role in the distribution of carbon sources in source and sink and the accumulation of carbohydrates in storage tissues. These findings will help to elucidate the genetic basis and regulatory mechanisms underlying starch properties in sweet potato.

## Materials and methods

### Plant material and growth conditions

The sweet potato variety Xushu22 (XS22) was cultivated at temperatures of between 22 and 28 °C in the experimental base of the Sweet Potato and Potato Research Institute, Southwest University, Chongqing, China. Leaf, stem, petiole, and root were sampled and diced at 95 days after transplanting (DAP) and quickly frozen in liquid nitrogen then stored at − 80 °C until use for RNA extraction. All *A. thaliana* and *N. benthamiana* plants were grown in a 22 °C and 28 °C climate chamber (16 h light/8 h dark) in Longping experimental building, Southwest University, Chongqing, China.

### Cloning sweet potato GPT genes and sequence analysis

To obtain the full-length mRNA sequences of target sweet potato GPT-encoding genes, the cDNAs of *GPT* genes were cloned using the SMARTer™ RACE cDNA amplification kit (Invitrogen, USA). RNA was extracted from the leaf, stem, petiole, and storage root of sweet potato variety Xushu 22 (XS22), and residual DNA was digested using the RNAprep Pure Plant Kit with DNase I (Tiangen Biotech, China) according to the manufacturer’s instructions. A 5-mg, equally proportioned (w/w) mixture of the above RNAs was used for first-strand cDNA synthesis. The gene-specific primers 83,665-5-1 (5′- GGTGTGTGCAACTGCAACTGGGAAGAGGG-3′) and 83,665-5-2 (5′- GCCTCACAGCCGAGATCATCATTAT-3′) were designed based on *IbG6PPT* transcripts [32, 36] and used to amplify the 5′ end of the *GPT* genes. The primers 83,665-3-1 (5′-GGTGGTTGCTCGCTTGCTGCTCTTACCG-3′) and 83,665-3-2 (5′-TCAGTATTGGAAACACCATGAAGCGT-3′) were used to amplify the 5′ and 3′ ends of *GPT* genes. PCR products were cloned into the pENTR-D-TOPO vector (Invitrogen, USA) and sequenced. Based on the obtained 5′- and 3′-end sequences of *GPT* genes, the full-length cDNA sequence was amplified using a 5′ primer (5′-ACACAACACACTGTACTTGTTTC-3′) and 3′ primer (5′-CAAAATTTGAAAGAGTTCCCTAACAG-3′) that were designed to match the 5′- and 3′-end sequences. PCR products were recombined into the Gateway entry vector pENTR-D-TOPO (Thermo Fisher, USA) for sequencing. Open reading frame (ORF) and sequence alignment was performed with Geneious Prime.

Transmembrane transport peptides were predicted by the TMPred tool in ExPASy (http://www.ch.embnet.org/software/TMPRED_form.html/, [14] and TMHMM (http://www.cbs.dtu.dk/services/TMHMM/) [9] using default parameters. Signal peptides were predicted by the SignalP 4.1 (http://www.cbs.dtu.dk/services/SignalP/) server using default parameters [22]. Conserved domains in the encoded proteins were analyzed with InterPro (http://www.ebi.ac.uk/interpro/) [26]. The three-dimensional structure of IbG6PPT1 was predicted using Swiss-Model (http://www.swissmodel.expasy.org), and the constructed model was examined and visualized with Chimera 1.2 (https://www.cgl.ucsf.edu/chimera/). Multiple sequence alignment results from ClustalW were used for phylogenetic tree construction by the neighbor-joining method with MEGAX [15]. Tree reliability was measured by bootstrap analysis with 1000 replicates.

### Expression pattern assay

The whole storage root of 95 DAP, 10 cm-length main stem, 5 cm-length petiole, and whole leaf of XS22 were sampled and diced. For each tissue, the diced samples were frozen in liquid nitrogen, grounded separately and then intensively mixed, and 0.1 g samples were used for RNA extraction. RNA (1 μg) extracted from the leaf, stem, petiole, and storage root was reverse transcribed in a 20 μL volume by the PrimeScript RT Master Mix (TaKaRa, China) according to the manufacturer’s instructions. The expression pattern of *GPT* genes was detected using primers and RT-qPCR methods as previously described [36]. Fold changes of the *GPT* transcripts were calculated according to the 2^–△△*Ct*^ method with three samples.

### Subcellular localization

The full coding sequence (CDS) of *IbG6PPT1* was cloned into pCAMBIA1300, and a GFP tag was fused to the C terminus of the gene. The empty vector was used as control. The constructs was transformed into *Agrobacterium tumefaciens* strain GV3101 (TransGen Biotech, China) and transiently expressed in *N. benthamiana* using syringe agroinfiltration [10]. GFP fluorescence was observed using a Zeiss LSM780 confocal laser scanning microscope (Zeiss, Germany; [19]). Signals were detected using excitation/emission wavelengths for GFP (488 nm/495–535 nm) and the chloroplast marker (633 nm/660–720 nm).

### Heterologous expression of IbG6PPT1 in *A. thaliana*

The full CDS of *IbG6PPT1* was recombined from the Gateway entry vector pENTR-D-TOPO (see the cloning and sequence analysis method above) into the destination vector pEarleyGate101 [6], yielding the construct *p35S*::*IbG6PPT1-YFP*, which has an N-terminal YFP tag. The construct *p35S*::*IbG6PPT1-YFP* was transformed into *A. thaliana* using the *Agrobacterium tumefaciens*–mediated floral dip method [4].

Positive transgenic lines were identified by PCR detection of *YFP* using the primers YFP-Fwd (5′-TGGTCGAGCTGGACGGCGACGTAAAC-3′) and YFP-Rev (5′-TTCTCGTTGGGGTCTTTGCTCAGGGC-3′) and by detection of the *bar* gene in the construct using the primers FBar (5′-TGGGCAGCCCGATGACAGCGACCAC-3′) and RBar (5′-ACCGAGCCGCAGGAACCGCAGGAGT-3′). *IbG6PPT1* expression in the transgenic *A. thaliana* plants was detected using the RT-qPCR method described in the expression pattern assay section. YFP expression was detected by western blotting using an anti-GFP antibody [29]. Thousand seed weight (g) was determined for 1000 seeds from each sample with three replicates.

### Starch and sugar measurement

The starch and soluble sugar contents of leaves and seeds in transgenic and control *A. thaliana* plants were determined using a previously published method [16]. The leaves and roots of 3-week-old seedlings were stained with an iodine solution (2% KI + 1% I_2_) and examined under a light microscope (Nikon, Japan), and images were captured using NIS-Elements BR 4.30.00 software as previously described [16].

## Supplementary Information

**Additional file 1: Table S1.** Quality trait in transgenic and control plants.

**Additional file 2: Figure 6b-1.** Original, uncropped western blot detection of IbG6PPT1 in Col-0, OX-14, OX-30, OX-76, OX-57. **Figure 6b-2.** Original, uncropped, grey background western blot detection of IbG6PPT1 in Col-0, OX-14, OX-30, OX-76, OX-57. **Figure 6b-3.** Original, uncropped ponceau staining of protein in Col-0, OX-14, OX-30, OX-76, OX-57.

## Data Availability

The datasets supporting the conclusions of this article are included within the article and its additional files. About proteins database could download from NCBI by their accession number.
