# Supplementary material for: Isolation and functional characterization of a glucose-6-phosphate/phosphate translocator (IbG6PPT1) from sweet potato (Ipomoea batatas (L.) Lam.)
Source: BMC Plant Biol. 2021 Dec 16;21:595. doi: 10.1186/s12870-021-03372-0 (PMC8675480; doi:10.1186/s12870-021-03372-0)
Supplement: Supplementary file 1 — Additional file 1: Table S1. Quality trait in transgenic and control plants. [file 12870_2021_3372_MOESM1_ESM.pdf]

Table S1 Quality trait in transgenic and control plants

Mean± SD	soluble sugar content of leaves(mg/g)	sample/ control	starch content of leaves(mg/g)	sample/ control	1000 seeds weight(g)	sample/ control	soluble sugar content of seeds(mg/g)	sample/ control	starch content of seeds(mg/g)	sample/ control
Col-0	0.5144±0.0675	100.00%	0.1680±0.0266	1.0000	0.0236±0.0020	1.0000	3.1394±0.1975	1.0000	3.1373±0.2326	1.0000
OX-14	0.4290±0.0397	83.40%	0.3226±0.0763	1.9209	0.0282±0.0002	1.1937	3.7816±0.1604	1.2046	4.0948±0.2524	1.3052
OX-30	0.4255±0.0390	82.70%	0.3115±0.0213	1.8544	0.0251±0.0003	1.0635	4.2269±0.0094	1.3464	3.8993±0.2706	1.2429
OX-76	0.3940±0.0285	76.59%	0.2770±0.0569	1.6491	0.0278±0.0002	1.1757	4.1922±0.1374	1.3353	3.9522±0.3622	1.2597
OX-57	0.4042±0.0280	78.58%	0.4612±0.0459	2.7459	0.0276±0.0007	1.1693	4.6040±0.0293	1.4665	3.5513±0.1304	1.1320
