# Supplementary figures and images for: Isolation and functional characterization of a glucose-6-phosphate/phosphate translocator (IbG6PPT1) from sweet potato (Ipomoea batatas (L.) Lam.)
Source: BMC Plant Biol. 2021 Dec 16;21:595. doi: 10.1186/s12870-021-03372-0 (PMC8675480; doi:10.1186/s12870-021-03372-0)

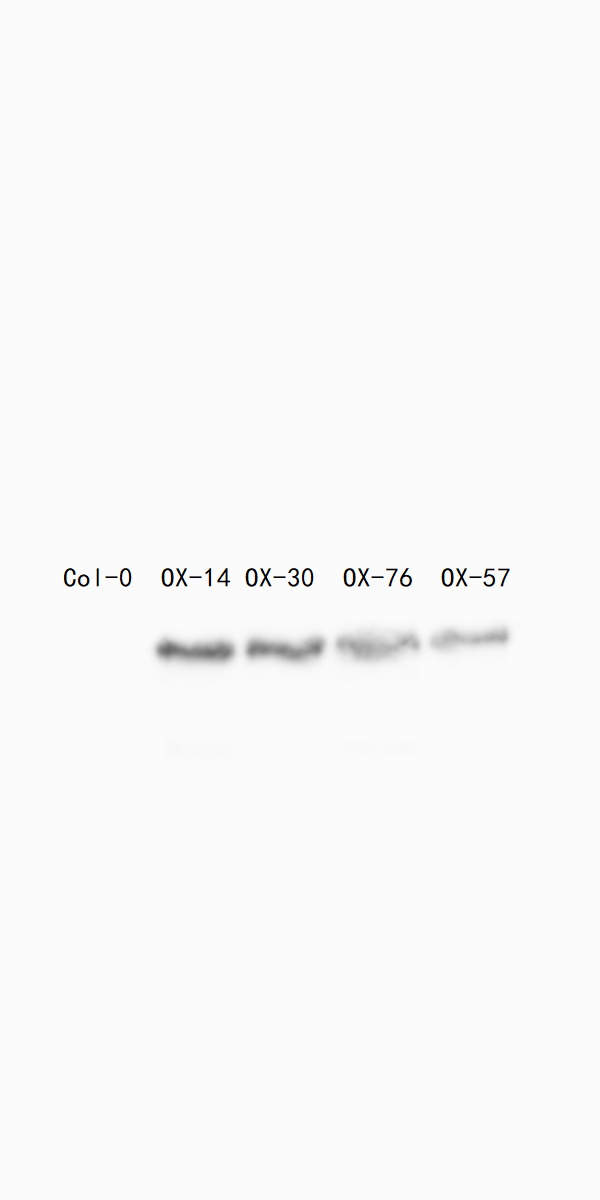

Supplement: Supplementary file 2 — Additional file 2: Figure 6b-1. Original, uncropped western blot detection of IbG6PPT1 in Col-0, OX-14, OX-30, OX-76, OX-57. Figure 6b-2. Original, uncropped, grey background western blot detection of IbG6PPT1 in Col-0, OX-14, OX-30, OX-76, OX-57. Figure 6b-3. Original, uncropped ponceau staining of protein in Col-0, OX-14, OX-30, OX-76, OX-57. [file 12870_2021_3372_MOESM2_ESM.zip › Figure 6b-1.tif]

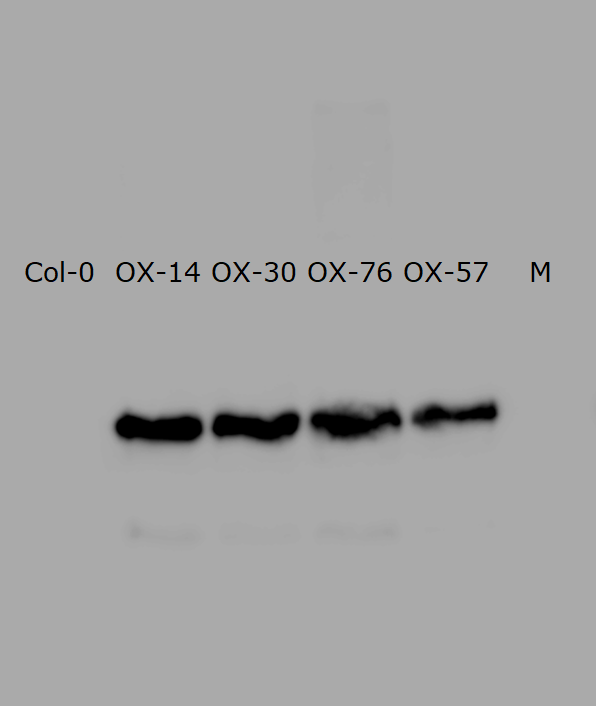

Supplement: Supplementary file 2 — Additional file 2: Figure 6b-1. Original, uncropped western blot detection of IbG6PPT1 in Col-0, OX-14, OX-30, OX-76, OX-57. Figure 6b-2. Original, uncropped, grey background western blot detection of IbG6PPT1 in Col-0, OX-14, OX-30, OX-76, OX-57. Figure 6b-3. Original, uncropped ponceau staining of protein in Col-0, OX-14, OX-30, OX-76, OX-57. [file 12870_2021_3372_MOESM2_ESM.zip › Figure 6b-2.tif]

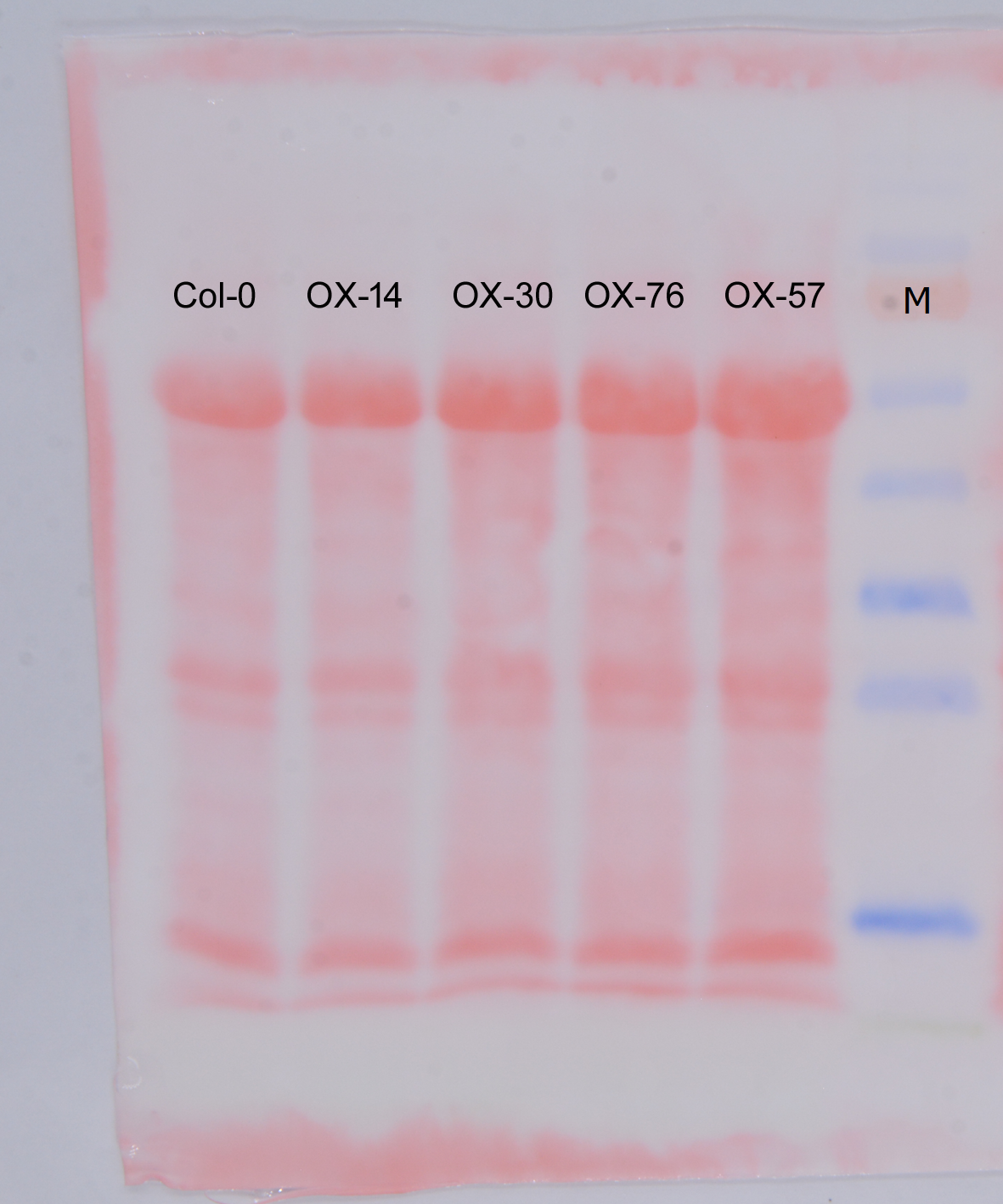

Supplement: Supplementary file 2 — Additional file 2: Figure 6b-1. Original, uncropped western blot detection of IbG6PPT1 in Col-0, OX-14, OX-30, OX-76, OX-57. Figure 6b-2. Original, uncropped, grey background western blot detection of IbG6PPT1 in Col-0, OX-14, OX-30, OX-76, OX-57. Figure 6b-3. Original, uncropped ponceau staining of protein in Col-0, OX-14, OX-30, OX-76, OX-57. [file 12870_2021_3372_MOESM2_ESM.zip › Figure 6b-3.tif]
